# Supplementary material for: IKKε isoform switching governs the immune response against EV71 infection
Source: Commun Biol. 2021 Jun 2;4:663. doi: 10.1038/s42003-021-02187-x (PMC8172566; doi:10.1038/s42003-021-02187-x)
Supplement: Supplementary file 2 — Supplementary Information [file 42003_2021_2187_MOESM2_ESM.pdf]

Supplementary Information

IKKε isoform switching governs the immune response against EV71 infection

Ya-Ling Chang[1,2], Yu-Wen Liao[1,2], Min-Hsuan Chen[2], Sui-Yuan Chang[1,3], Yao-Ting Huang[4,8], Bing-Ching Ho[2,8], Sung-Liang Yu[1,2,3,5,6,7,8]

1 Department of Clinical Laboratory Sciences and Medical Biotechnology, College of Medicine, National Taiwan University, Taipei, Taiwan
2 Centers of Genomic and Precision Medicine, National Taiwan University, Taipei, Taiwan
3 Department of Laboratory Medicine, National Taiwan University Hospital, Taipei, Taiwan
4 Department of Computer Science and Information Engineering, National Chung Cheng University, Chia-Yi, Taiwan
5 Institute of Medical Device and Imaging, College of Medicine, National Taiwan University, Taipei, Taiwan
6 Graduate Institute of Pathology, College of Medicine, National Taiwan University, Taipei, Taiwan
7 Graduate Institute of Clinical Medicine, National Taiwan University College of Medicine, Taipei, Taiwan

Section details	Title	Page
**Methods**	Droplet digital PCR analysis	3
**Tables**		
Supplementary Table 1	Enriched pathways with isoform switching genes in EV71 infection	4
Supplementary Table 2	List of primers and probes	5
Supplementary Table 3	The raw data of ddPCR	6
**Figures**		
Supplementary Figure 1	The pathway enriched by isoform switching genes in EV71 infection	7
Supplementary Figure 2	IKKε v2 transcriptionally induces ISGs expressions in EV71 infection through IRF7	10
Supplementary Figure 3	EV71 infection promotes ubiquitination	12
Supplementary Figure 4	IKKε v2 increases IRF7 phosphorylation and IRF7 translocation in the presence of ubiquitin	14
Supplementary Figure 5	The interaction of IKK isoforms and IRF7	17
Supplementary Figure 6	The expression levels of HA-ubiquitin, referred to Figure 5	19
Supplementary Figure 7	Full-scan images of representative Western blots, reffered to Figure 1	22
Supplementary Figure 8	Full-scan images of representative Western blots, reffered to Figure 2	23
Supplementary Figure 9	Full-scan images of representative Western blots, reffered to Figure 3	24
Supplementary Figure 10	Full-scan images of representative Western blots, reffered to Figure 4	25
Supplementary Figure 11	Full-scan images of representative Western blots, reffered to Figure 5	26
Supplementary Figure 12	Full-scan images of representative Western blots, reffered to Supplementary Figure 2	28
Supplementary Figure 13	Full-scan images of representative Western blots, reffered to Supplementary Figure 3	29
Supplementary Figure 14	Full-scan images of representative Western blots, reffered to Supplementary Figure 4	30
Supplementary Figure 15	Full-scan images of representative Western blots, reffered to Supplementary Figure 5	31
Supplementary Figure 16	Full-scan images of representative Western blots, reffered to Supplementary Figure 6	32

Supplementary Methods

Droplet digital PCR analysis

Three commercially available probes purchased from Thermo Fisher were used to detect IKKε isoforms in which Hs01063858-m1 detects all the three IKKε isoforms, Hs01069870_m1 detects both IKKε v1 and v2 and Hs01063855_g1 detects both IKKε v1 and v3. After ddPCR assays, we subtracted copies of Hs01069870_m1 (detecting v1 and v2) from Hs01063858-m1 (detecting v1, v2, and v3) to have IKKε v3. Similarly, we subtracted copies of Hs01063855_g1 (detecting v1 and v3) from Hs01063858-m1 (detecting v1, v2, and v3) to have IKKε v2. Finally, copies of Hs01063858-m1 minus IKKε v3 and IKKε v2 leaves IKKε v1. Ratio of individual IKKε isoform was calculated by copy number of each IKKε isoform divided by total copies of three isoforms.

Supplementary Table 1. Enriched pathways with isoform switching genes in EV71 infection

Rank	Pathway	Entities	Genes	*p* value
1	Toll-like receptor signaling pathway	6	IRAK4, AKT1, IKBKE, MYD88, IRF3, SPP1	0.022
2	PPAR signaling pathway	5	PPARD, DBI, SCP2, ACAA1, ANGPTL4	0.024
3	Neurotrophin signaling pathway	6	IRAK4, AKT1, BDNF, CAMK2B, SH2B1, TP73	0.047
4	Apoptosis	5	IRAK4, AKT1, TNFRSF10B, MYD88, IL1RAP	0.049

Supplementary Table 2. List of primers and probes

Gene name	Sequence (5’-3’)
IFNb-SYBR-F	ATTGCCTCAAGGACAGGATG
IFNb-SYBR-R	GGCCTTCAGGTAATGCAGAA
OAS1-SYBR-F	CCCCATTATTGAAAAGTACCTGAGA
OAS1-SYBR-R	GCCGGGTCCAGGATCAC
ISG56-SYBR-F	CAGAACGGCTGCCTAATTTACA
ISG56-SYBR-R	GTGGGTCCTGCTTTTTCTCTGT
ISG20-SYBR-F	CCCTGCGGGTGCTGAGT
ISG20-SYBR-R	TGTCCAAGCAGGCTGTTCTG
Mxa-SYBR-F	GCT ACTGTGGCCCAGAAAAATC
Mxa-SYBR-R	TCATACTGGCTGCACAGGTTGT
**Promoter Luc reporter**	**Sequence (5'-3')**
OAS1-F	CCCGGTACCCTTAACAAAAAGAAAAGAGAC
OAS1-R	TTTAAGCTTTTTACCACCTTGGACACACA
ISG56-F	TAAGGTACCGCACCCAGCCAAGAATCATT
ISG56-R	CGCAAGCTTAGATCTGGCTATTCTGTCTT
ISG20-F	AAAGGTACCCCAAATCCCACTTGGTGAAA
ISG20-R	AAAAAGCTTCTCTCACCTGCCTGCCTCTG
Mxa-F	ACCGGTACCCCAAAGCTCACCAGTATCAA
Mxa-R	ATAAAGCTTCTCTGCTACCAGGCTGAGGA
**Expression vector**	**Sequence (5'-3')**
IRF7-HindIII-F	AAAAAGCTTATGGCCTTGGCTCCTGAGAGGGCAG
IRF7-BamHI-R	AAAGGATCCGGCGGGCTGCTCCAGCTCCATAAGG
**Digital Probe**	**Target exon boundary**
Hs01063858_m1	NM_014002.3 Exon 21-22
Hs01069870_m1	NM_014002.3 Exon 2-3
Hs01063855_g1	NM_014002.3 Exon 19-20

Supplementary Table 3. The raw data of ddPCR

Sample	Probes		
	Hs01063858	Hs01069870	Hs01063855
RD_Mock infection	3330	2960	2780
RD_4 h.p.i.	5140	4450	3820
RD_8 h.p.i.	1750	1436	1026
SH-SY5Y_Mock infection	2620	2120	2440
SH-SY5Y_12 h.p.i.	3260	2120	2900
SH-SY5Y_24 h.p.i.	2440	1656	2140
	Copies of each IKKε		
	IKKε v1	IKKε v2	IKKε v3
RD_Mock infection	2410	550	370
RD_4 h.p.i.	3130	1320	690
RD_8 h.p.i.	712	724	314
SH-SY5Y_Mock infection	1940	180	500
SH-SY5Y_12 h.p.i.	1760	360	1140
SH-SY5Y_24 h.p.i.	1356	300	784
	Ratio of each IKKε		
	IKKε v1	IKKε v2	IKKε v3
RD_Mock infection	72%	17%	11%
RD_4 h.p.i.	61%	26%	13%
RD_8 h.p.i.	41%	41%	18%
SH-SY5Y_Mock infection	74%	7%	19%
SH-SY5Y_12 h.p.i.	54%	11%	35%
SH-SY5Y_24 h.p.i.	56%	12%	32%

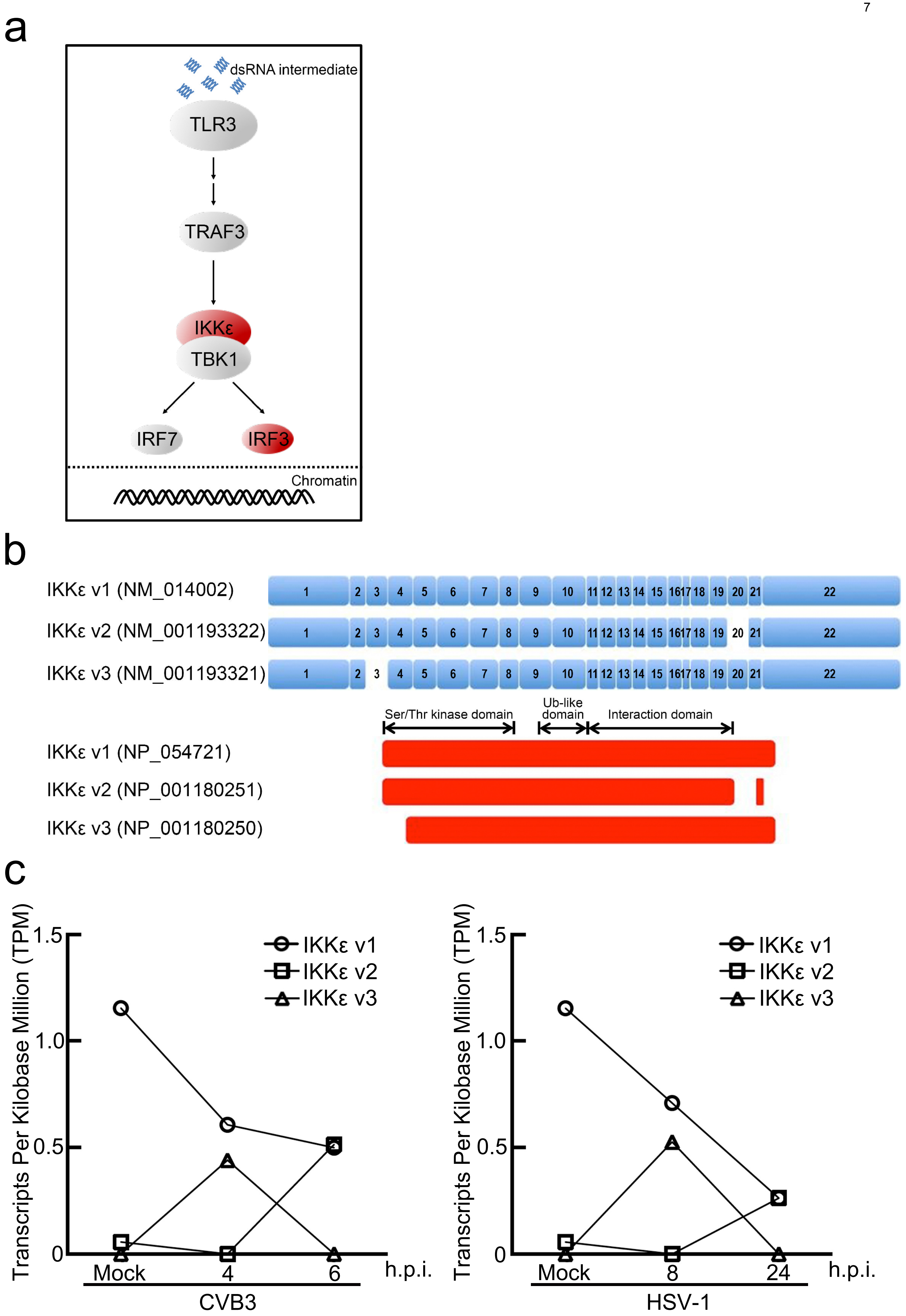
a
dsRNA intermediate
TLR3
TRAF3
IKKε
TBK1
IRF7
IRF3
Chromatin
b
IKKε v1 (NM_014002)
IKKε v2 (NM_001193322)
IKKε v3 (NM_001193321)
Ser/Thr kinase domain
Ub-like domain
Interaction domain
IKKε v1 (NP_054721)
IKKε v2 (NP_001180251)
IKKε v3 (NP_001180250)
c
Transcripts Per Kilobase Million (TPM)
IKKε v1
IKKε v2
IKKε v3
Mock
4
6
h.p.i.
CVB3
Mock
8
24
h.p.i.
HSV-1

Supplementary Fig. 1: The pathway enriched by isoform switching genes in EV71 infection.

a. Toll-like receptor signaling pathway enriched in EV71 infection. The isoform switching genes identified in EV71 infection were adapted to DAVID Bioinformatics Resources analysis and the Toll-like receptor signaling pathway was ranked as top-one pathway with most significance (*p* value= 0.021). The illustrated pathway is a part of Toll-like receptor signaling pathway referenced from DAVID Bioinformatics Resources. The red circle and the gray circle represented the gene with and without isoform switching in EV71 infection, respectively.

b. Illustration of IKKε isoforms. IKKε gene possesses three isoforms: isoform 1 (IKKε v1, NM_014002; NP_054721) contains full-length coding DNA sequence (CDS) while v2 (IKKε v2, NM_001193322; NP_001180251) and v3 (IKKε v3, NM_001193321; NP_001180250) truncates exon 20 and exon 3, respectively. In protein level, IKKε v2 is defected in coiled-coil domain functioned as protein-protein interaction and IKKε v3 is trimmed in kinase domain contrast to IKKε v1.

c. IKKε isoform switching is measured by RNA-Seq in CVB3 and HSV-1 infected HeLa cells. IKKε v2 was up-regulated while IKKε v1 was down-

regulated in response to CVB3 (left panel) and HSV-1 (right panel) infection determined by RNA-Seq.

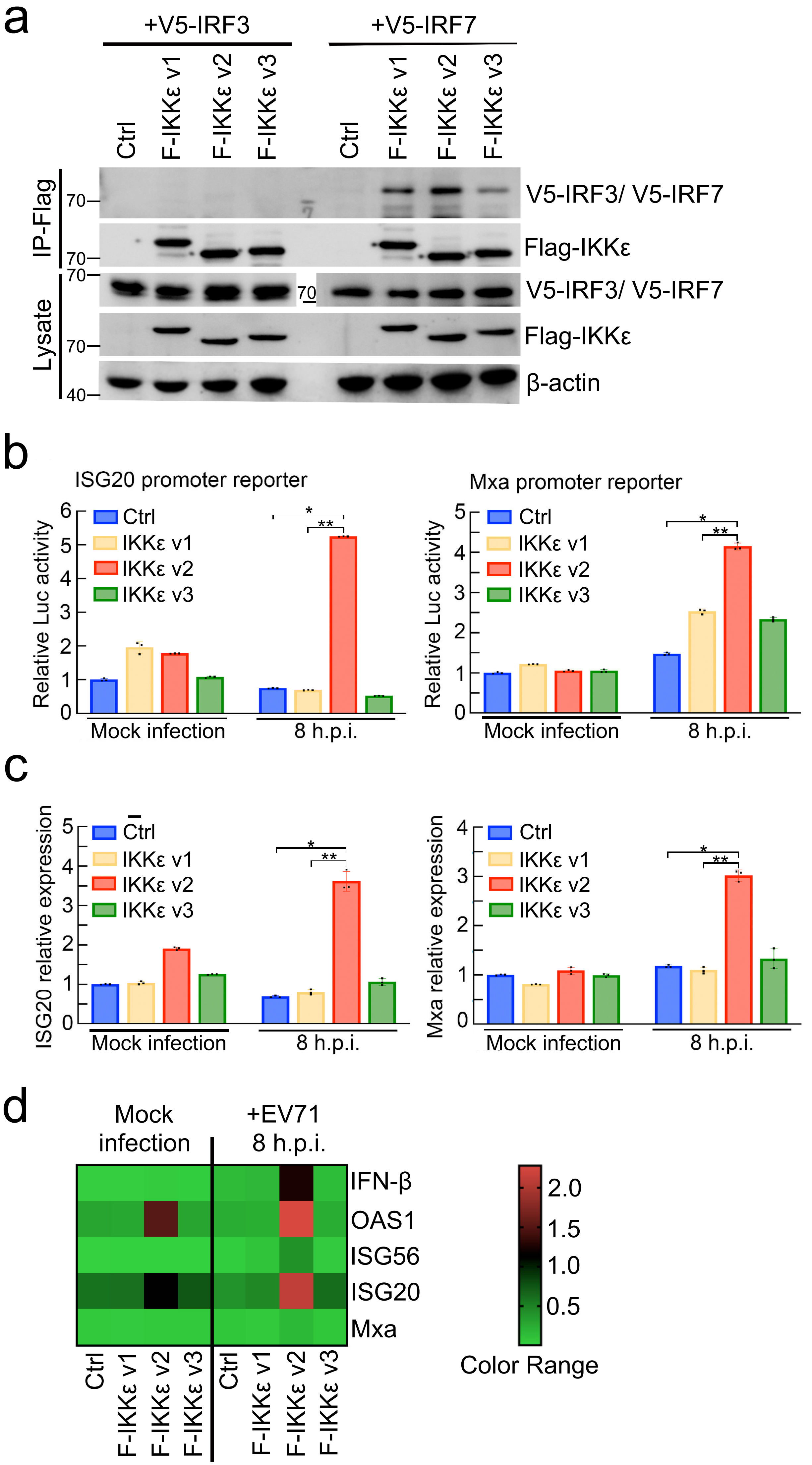
a
+V5-IRF3
+V5-IRF7
Ctrl
F-IKKε v1
F-IKKε v2
F-IKKε v3
IP-Flag
Lysate
70
40
V5-IRF3/ V5-IRF7
Flag-IKKε
β-actin
b
ISG20 promoter reporter
Mxa promoter reporter
Relative Luc activity
Ctrl
IKKε v1
IKKε v2
IKKε v3
Mock infection
8 h.p.i.
c
ISG20 relative expression
Mxa relative expression
d
Mock infection
+EV71 8 h.p.i.
IFN-β
OAS1
ISG56
ISG20
Mxa
2.0
1.5
1.0
0.5
Color Range

Supplementary Fig. 2: IKKε v2 transcriptionally induces ISGs expressions in EV71 infection through IRF7.

a. IKKε preferentially binds to IRF7. Each Flag-IKKε isoform was co-transfected with V5-IRF3 or V5-IRF7 in HEK293T cells. Flag-IKKε was immunoprecipitated with anti-Flag beads. V5-IRF3 (left panel) and V5-IRF7 (right panel) were detected by anti-V5 antibody.

b, c. IKKε v2 up-regulates promoter activities and expressions of MxA and ISG20. Each IKKε isoform was ectopically expressed in RD cells accompanied with the indicated ISG promoter followed by EV71 infection. The promoter activities (b) and ISGs expressions (c) were measured at indicated h.p.i.. The data are normalized with vector control (Ctrl) in mock infection group. * and ** represent p value <0.05 as compared with Ctrl group and IKKε v1 group, respectively. All data presented are mean ± SD (n=3).

d. The heat map of ISGs expressions in ectopically IKKε isoform-expressed RD cells, referred to Fig. 2b, 2d, S2a and S2b. The color scale corresponds to the mean for each gene.

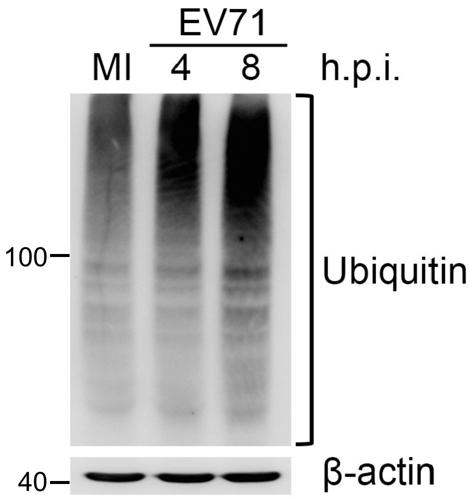
EV71
MI
4
8
h.p.i.
100
Ubiquitin
40
β-actin

Supplementary Fig. 3: EV71 infection promotes ubiquitination.

RD cells were infected with EV71 at 10 m.o.i. and harvested at indicated h.p.i.. The total lysates were adapted to immunoblot analysis with anti-ubiquitin antibody. β-actin was served as an internal control. MI: mock infection

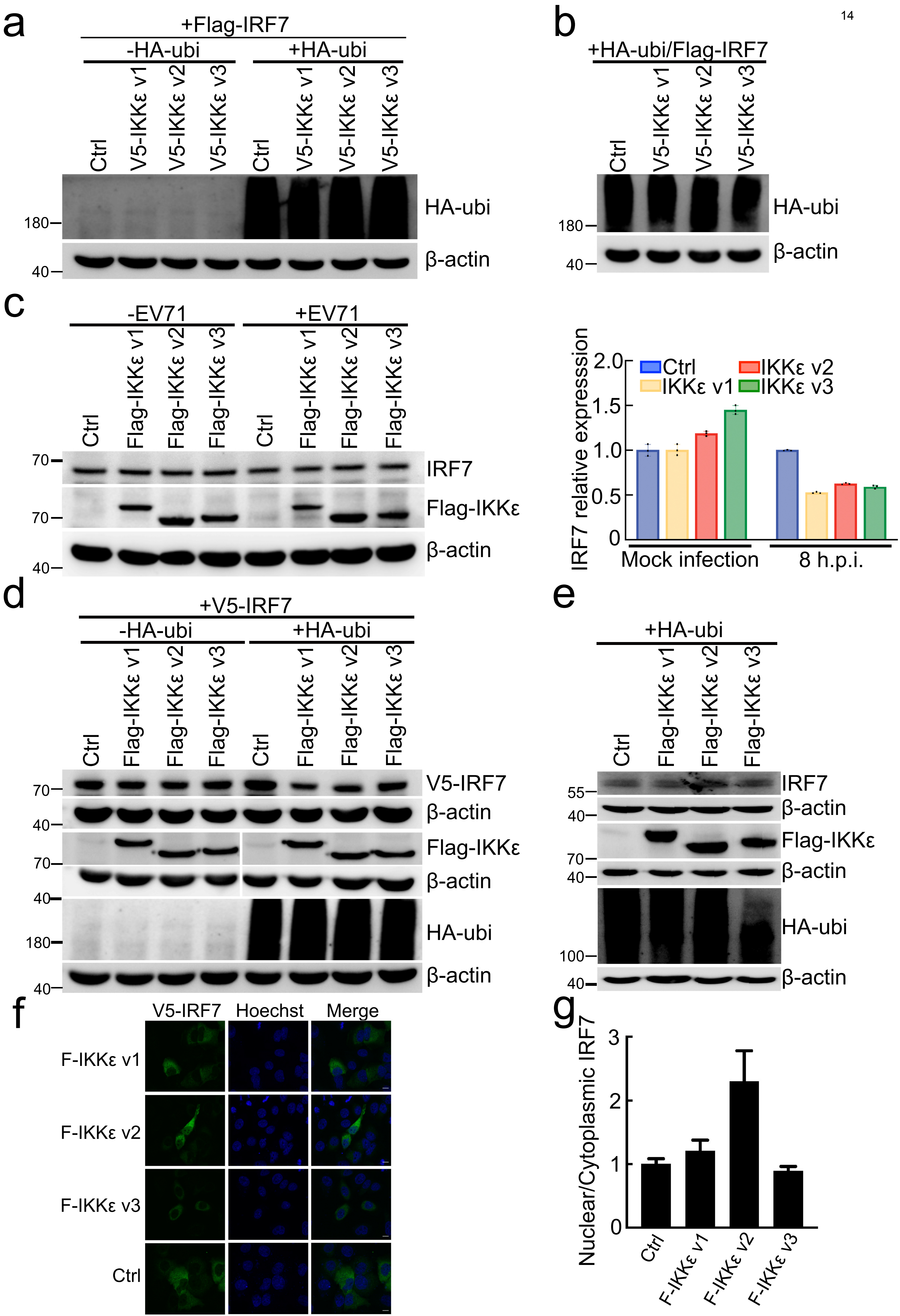
a
+Flag-IRF7
-HA-ubi
+HA-ubi
Ctrl
V5-IKKε v1
V5-IKKε v2
V5-IKKε v3
HA-ubi
β-actin
180
40
b
+HA-ubi/Flag-IRF7
c
-EV71
+EV71
Flag-IKKε v1
Flag-IKKε v2
Flag-IKKε v3
IRF7
Flag-IKKε
70
IRF7 relative expression
Ctrl
IKKε v1
IKKε v2
IKKε v3
Mock infection
8 h.p.i.
d
+V5-IRF7
V5-IRF7
e
55
100
f
V5-IRF7
Hoechst
Merge
F-IKKε v1
F-IKKε v2
F-IKKε v3
g
Nuclear/Cytoplasmic IRF7

Supplementary Fig. 4: IKKε v2 increases IRF7 phosphorylation and IRF7 translocation in the presence of ubiquitin.

a, b. The expression levels of HA-ubiquitin referred to Fig. 3a, 3b. HEK293or HeLa cells were transfected with indicated plasmids and total lysates were loaded to perform immunoblot with indicated antibodies. β-actin served as an internal control.

c. IRF7 mRNA and protein were not induced by IKKε v2. RD cells were ectopically expressed Flag- IKKε followed by EV71 infection. Total lysates were loaded to perform immunoblot with indicated antibodies. β-actin served as an internal control (left panel). RNA expression of IRF7 was measured by quantitative real-time PCR and normalized with Ctrl in mock infection group (right panel). All data presented are mean ± SD (n=3).

d, e. The expression levels of HA-ubiquitin referred to Fig. 3d, 3e. HeLa cells were transfected with indicated plasmids and total lysates were loaded to perform immunoblot with indicated antibodies. β-actin served as an internal control.

f. IKKε v2 facilitates IRF7 translocation by immunofluorescence analysis. The immunofluorescence images of cells treated with the same condition used in Figure 3d. Scale bar: 10um

g. The ratio of nuclear IRF7 to cytoplasmic IRF7 in Supplementary Figure 3f. The ratios of F- IKKε isoforms were normalized to that of Ctrl.

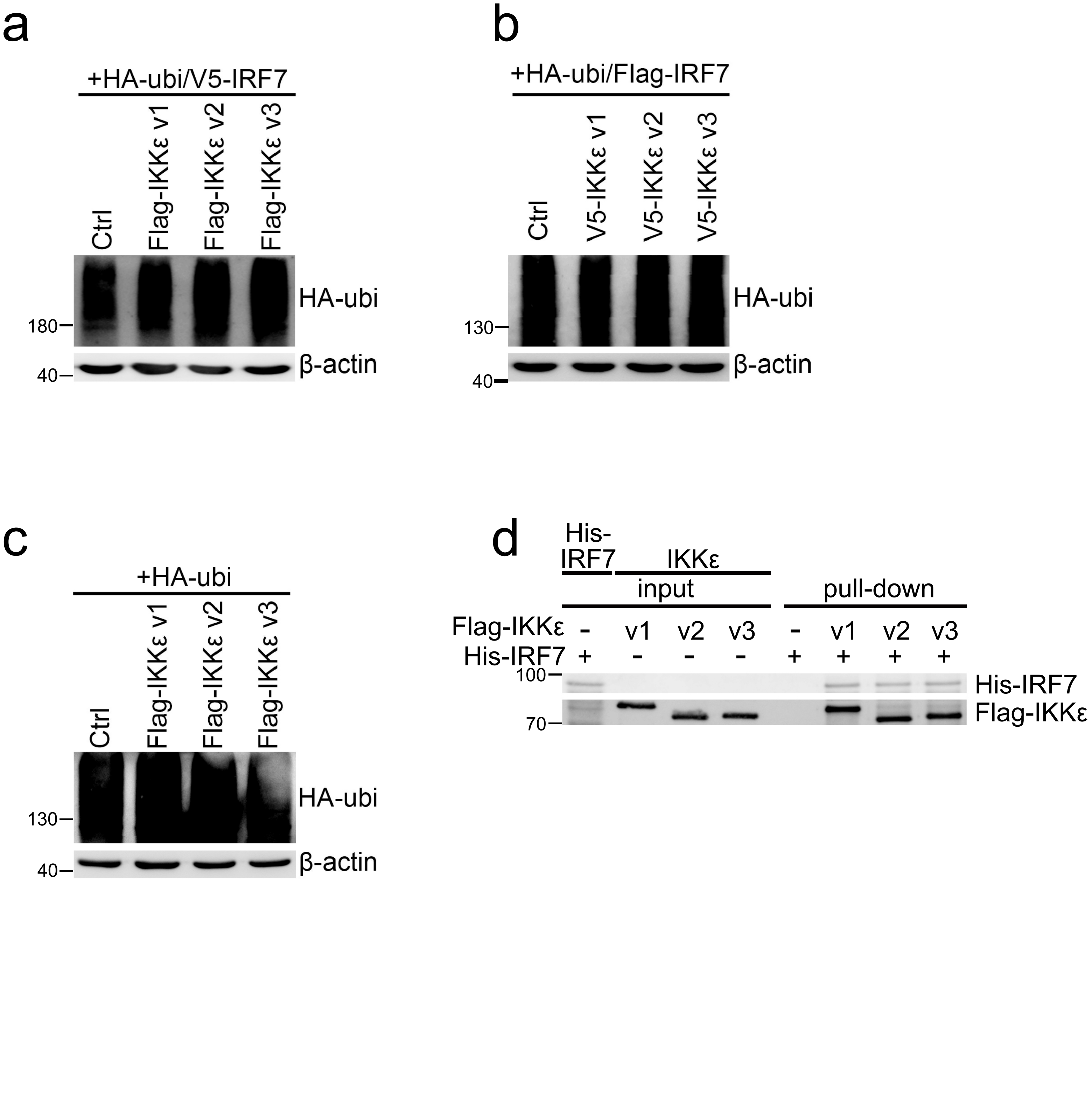
a
+HA-ubi/V5-IRF7
Ctrl
Flag-IKKε v1
Flag-IKKε v2
Flag-IKKε v3
HA-ubi
180
β-actin
40
b
+HA-ubi/Flag-IRF7
Ctrl
V5-IKKε v1
V5-IKKε v2
V5-IKKε v3
HA-ubi
130
β-actin
40
c
+HA-ubi
Ctrl
Flag-IKKε v1
Flag-IKKε v2
Flag-IKKε v3
HA-ubi
130
β-actin
40
d
His-IRF7
IKKε
input
pull-down
Flag-IKKε - v1 v2 v3 - v1 v2 v3
His-IRF7 + - - - + + + +
100
His-IRF7
70
Flag-IKKε

Supplementary Fig. 5: The interaction of IKK isoforms and IRF7.

a-c. The expression levels of HA-ubiquitin, referred to Fig. 4. HEK293 cells were transfected with indicated expressing plasmids and total lysates were loaded to perform immunoblot with anti-HA antibody. β-actin served as an internal control.

d. The direct interaction of IRF7 and IKKε isoforms. Each Flag-IKKε isoform was purified by anti-Flag beads from Flag-IKKε-expressed HEK293T cells and incubated with 1 μg of His-IRF7, which was purified from *E. coli.* for 2 hours at 4°C. After washing for three times, the bound His-IRF7 was analyzed by Western blotting with anti-His antibody.

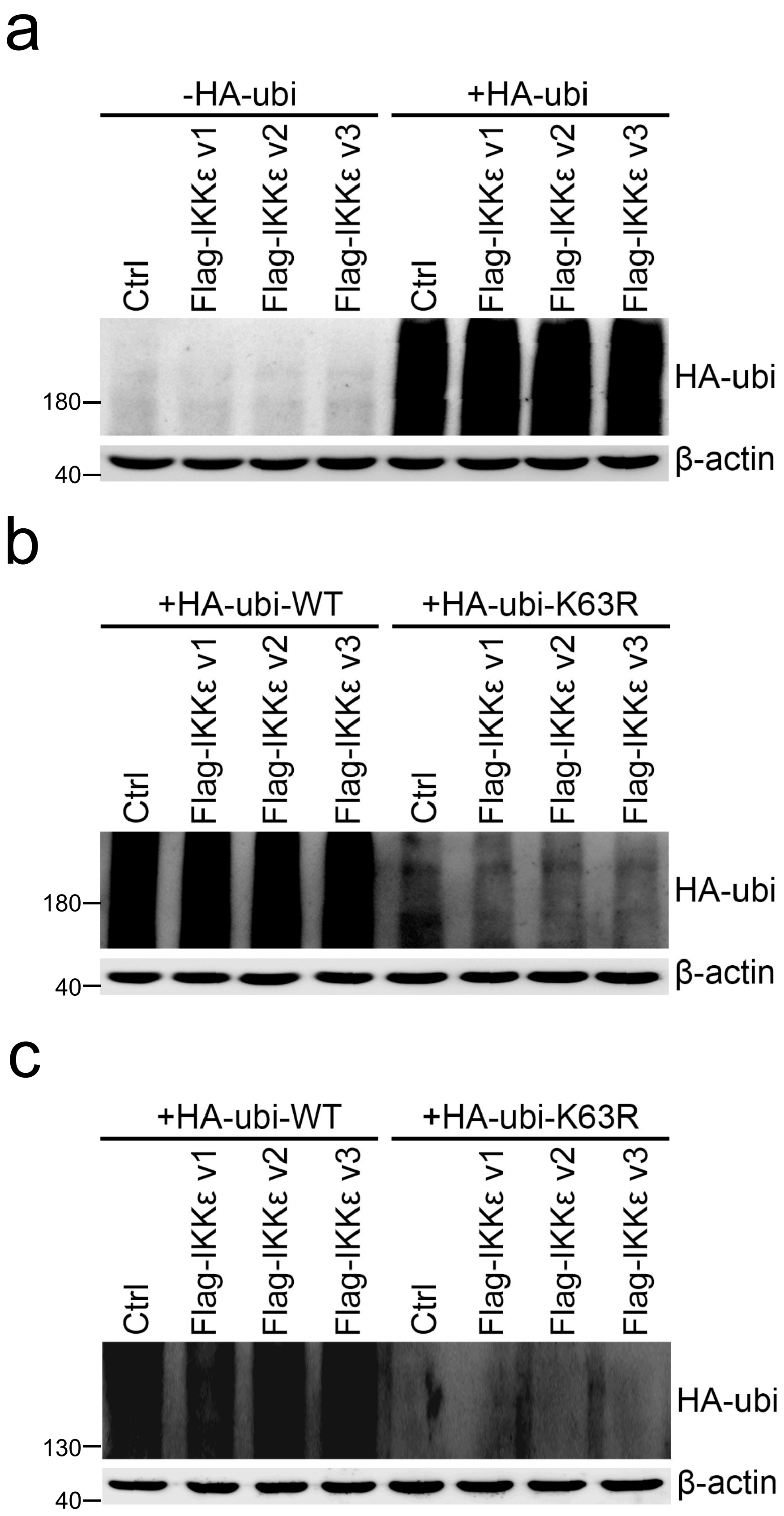
a
-HA-ubi
+HA-ubi
Ctrl
Flag-IKKε v1
Flag-IKKε v2
Flag-IKKε v3
HA-ubi
β-actin
180
40
b
+HA-ubi-WT
+HA-ubi-K63R
Ctrl
Flag-IKKε v1
Flag-IKKε v2
Flag-IKKε v3
HA-ubi
β-actin
180
40
c
+HA-ubi-WT
+HA-ubi-K63R
Ctrl
Flag-IKKε v1
Flag-IKKε v2
Flag-IKKε v3
HA-ubi
β-actin
130
40

Supplementary Fig. 6: The expression levels of HA-ubiquitin, referred to Figure 5.

a-c. HEK293 cells were transfected with indicated expressing plasmids and total lysates were loaded to perform immunoblot with anti-HA antibody. β-actin served as an internal control.

Supplementary Figure 7-11: Full-scan images of representative Western blots, reffered to Figure 1-5

1c

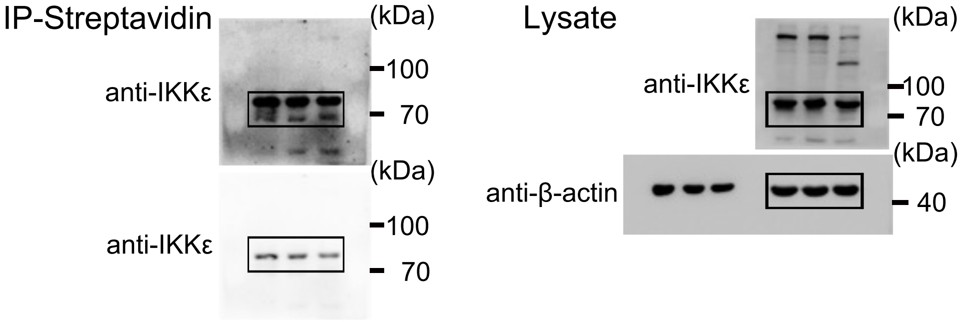
IP-Streptavidin
(kDa)
anti-IKKε
100
70
(kDa)
anti-IKKε
100
70
Lysate
(kDa)
anti-IKKε
100
70
(kDa)
anti-β-actin
40

2f

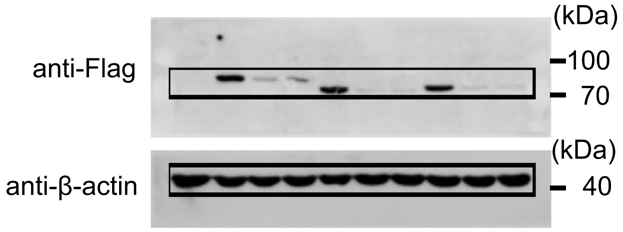

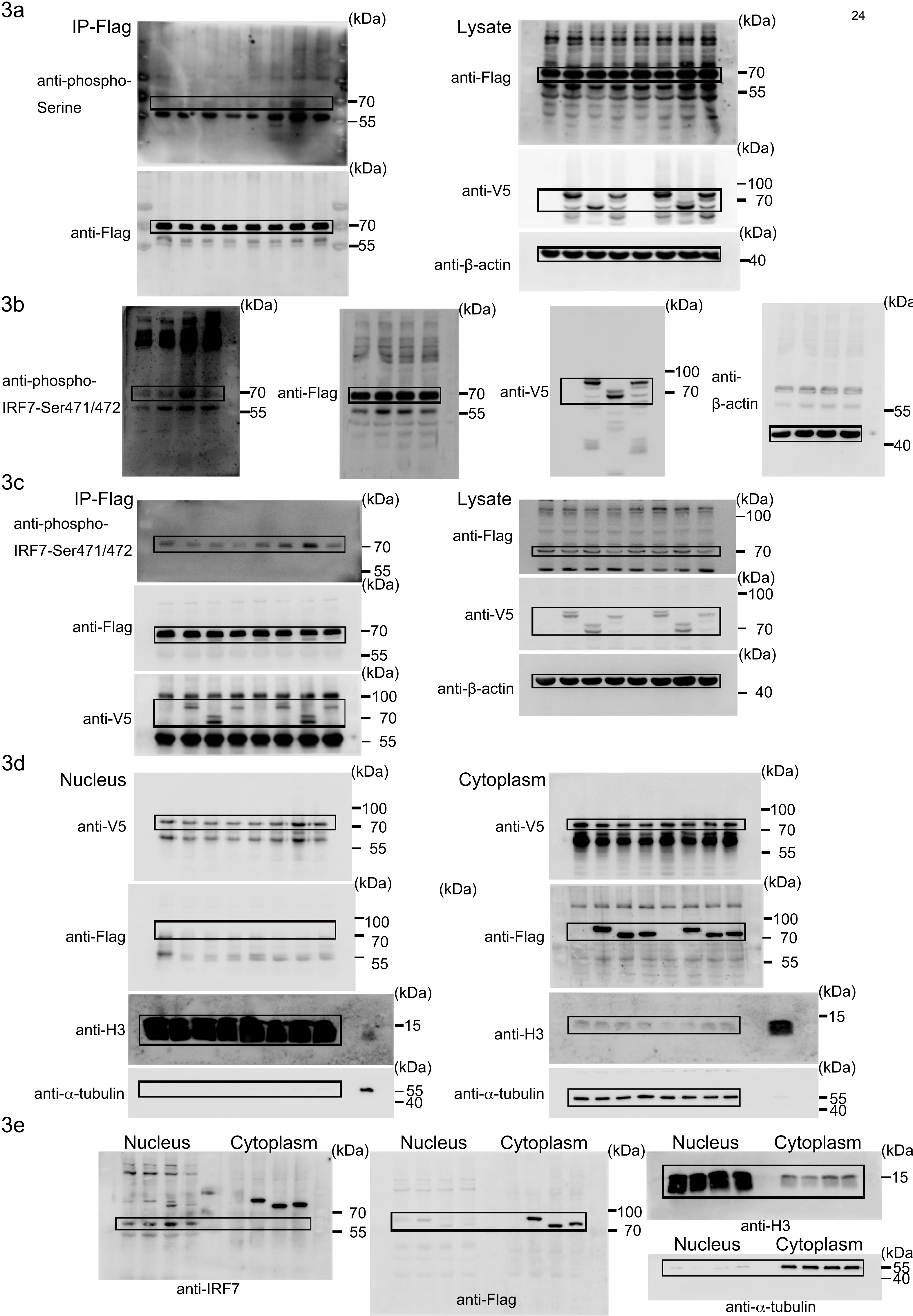
3a
IP-Flag
anti-phospho-
Serine
anti-Flag
Lysate
anti-Flag
anti-V5
anti-β-actin
(kDa)
100
70
55
40
3b
anti-phospho-
IRF7-Ser471/472
anti-Flag
anti-V5
anti-
β-actin
3c
IP-Flag
anti-phospho-
IRF7-Ser471/472
anti-Flag
anti-V5
Lysate
anti-Flag
anti-V5
anti-β-actin
3d
Nucleus
anti-V5
anti-Flag
anti-H3
anti-α-tubulin
Cytoplasm
anti-V5
anti-Flag
anti-H3
anti-α-tubulin
15
3e
Nucleus
Cytoplasm
anti-IRF7
anti-Flag
anti-H3
anti-α-tubulin

4a

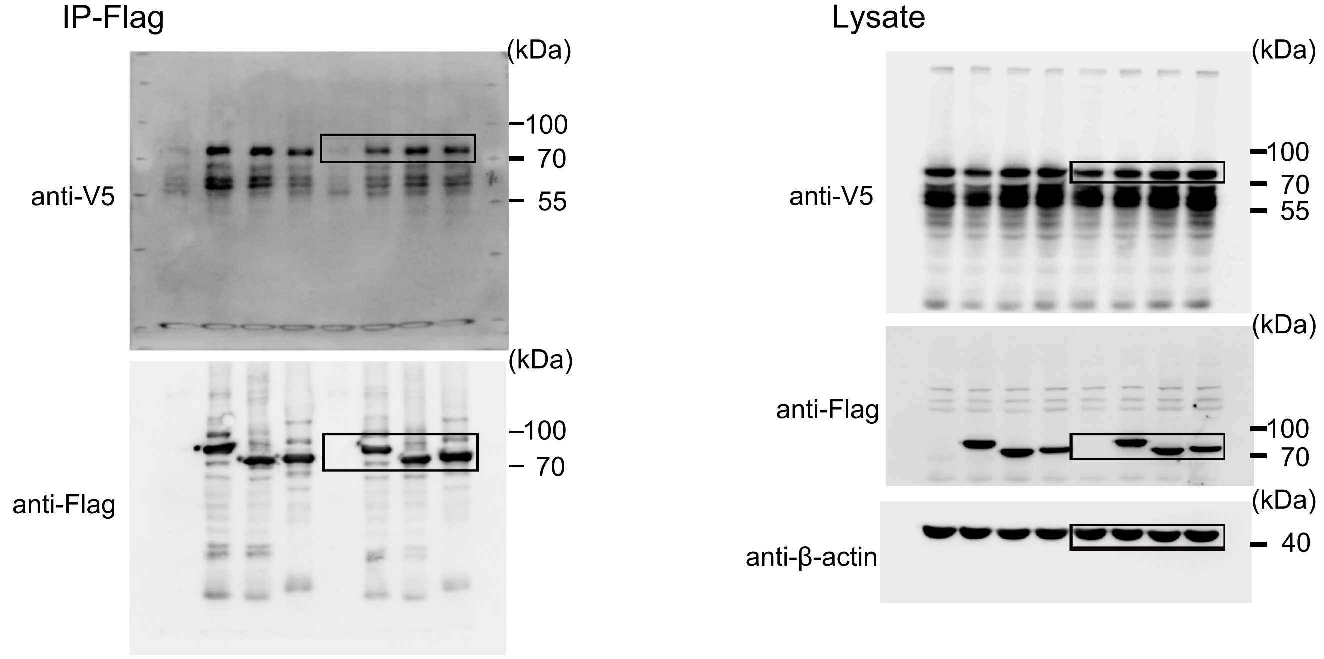
IP-Flag
Lysate
(kDa)
anti-V5
anti-Flag
anti-β-actin
100
70
55
40

4b

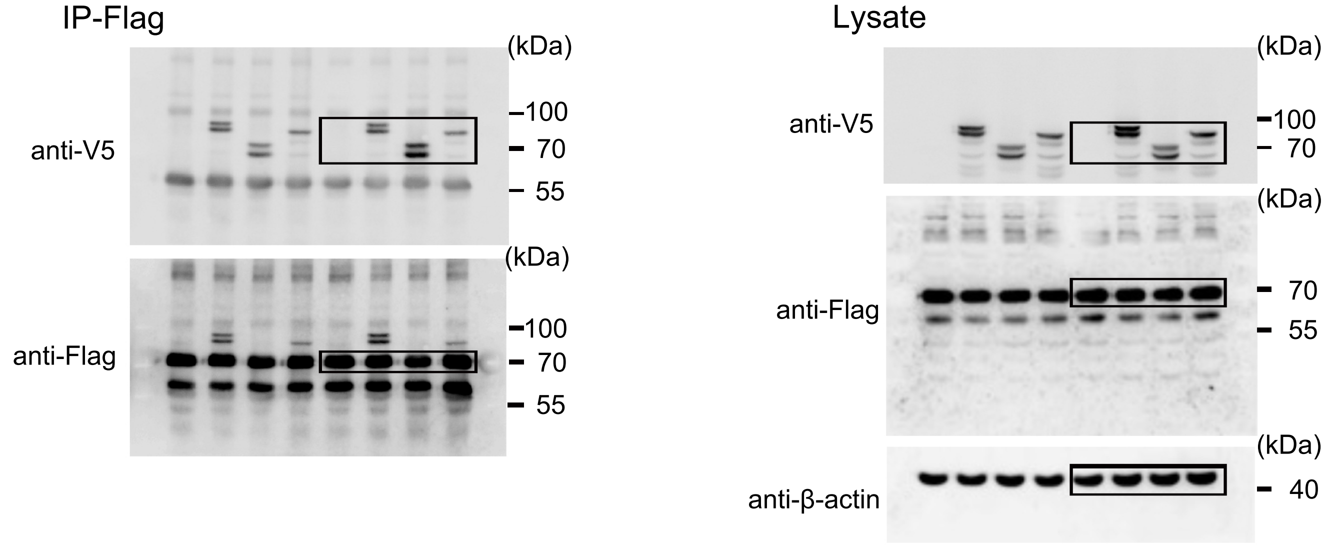
IP-Flag
Lysate
(kDa)
anti-V5
anti-Flag
anti-β-actin
100
70
55
40

4c

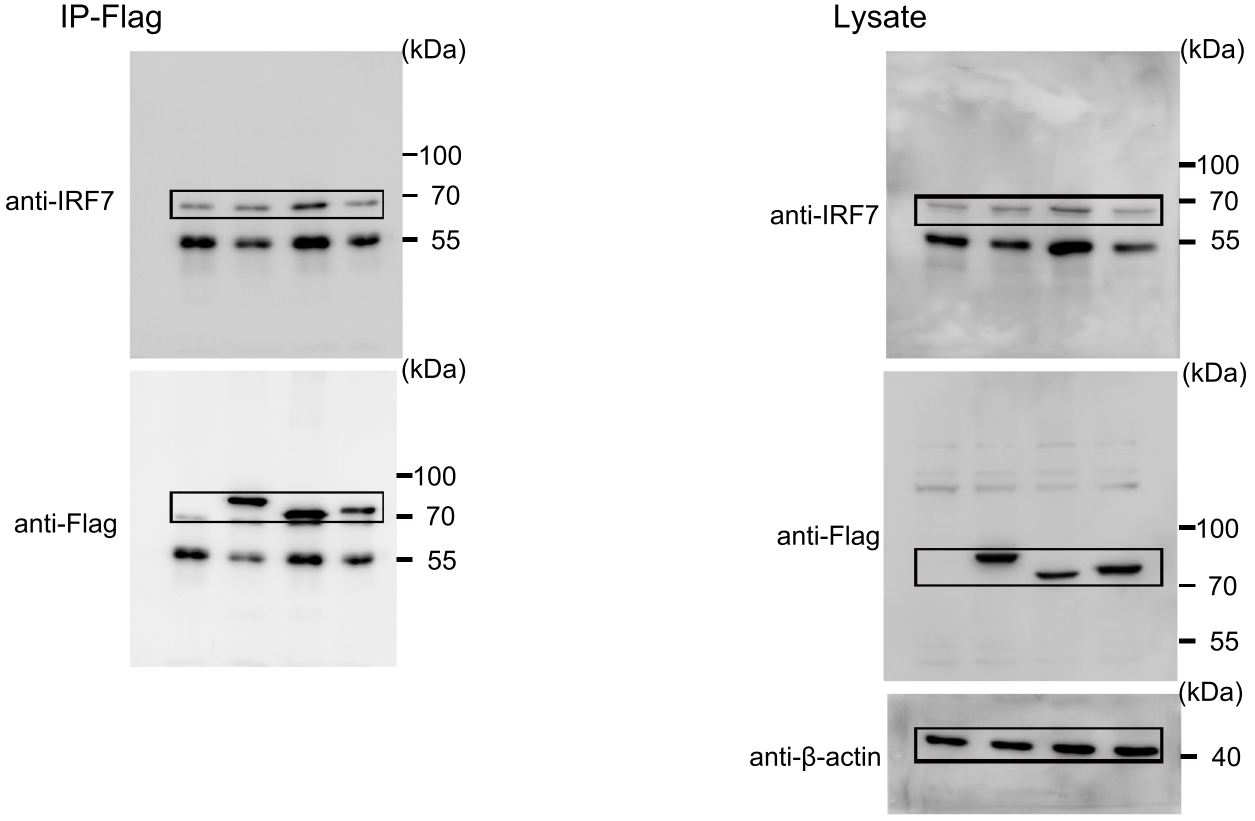
IP-Flag
Lysate
(kDa)
anti-IRF7
anti-Flag
anti-β-actin
100
70
55
40

5a

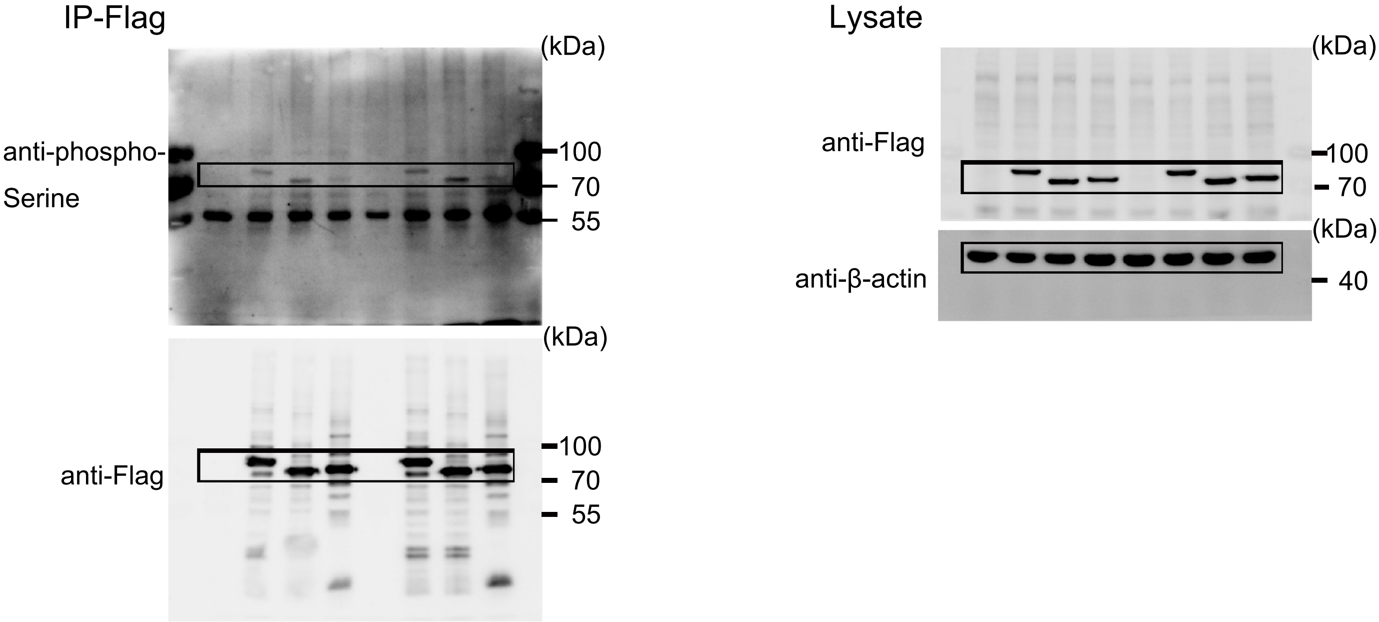
IP-Flag
(kDa)
anti-phospho-
Serine
100
70
55
(kDa)
anti-Flag
100
70
55
Lysate
(kDa)
anti-Flag
100
70
(kDa)
anti-β-actin
40

5b

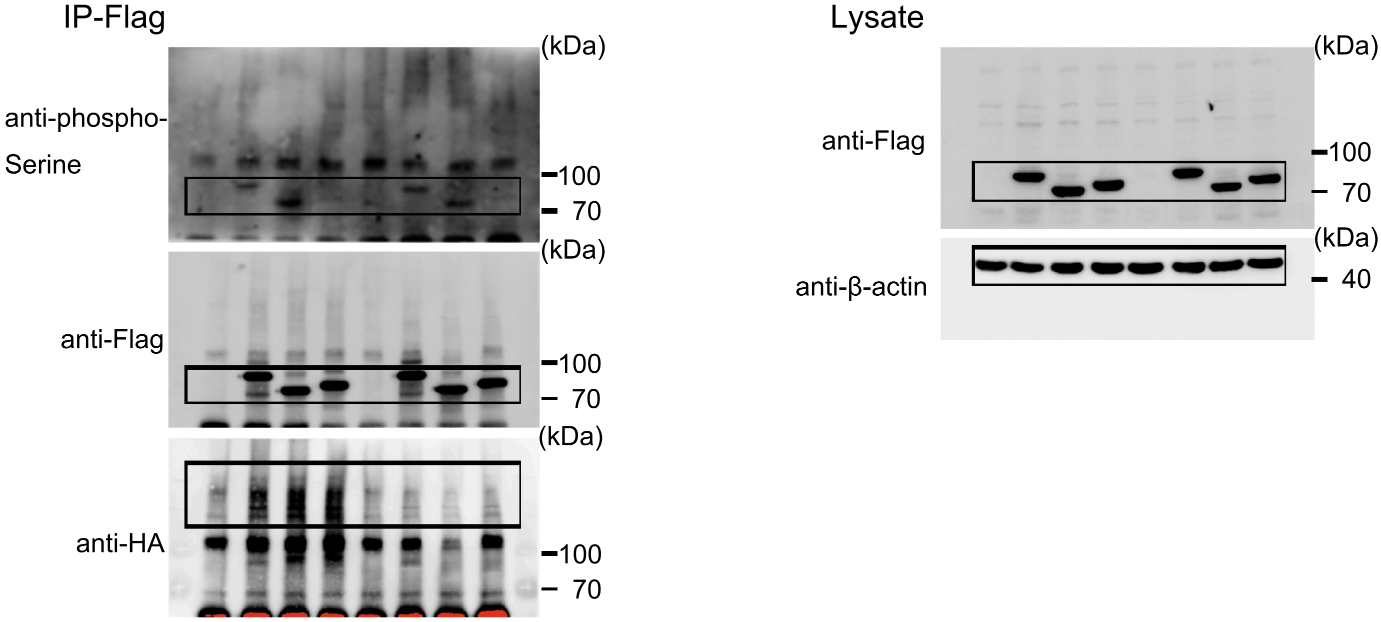
IP-Flag
(kDa)
anti-phospho-
Serine
100
70
(kDa)
anti-Flag
100
70
(kDa)
anti-HA
100
70
Lysate
(kDa)
anti-Flag
100
70
(kDa)
anti-β-actin
40

5c

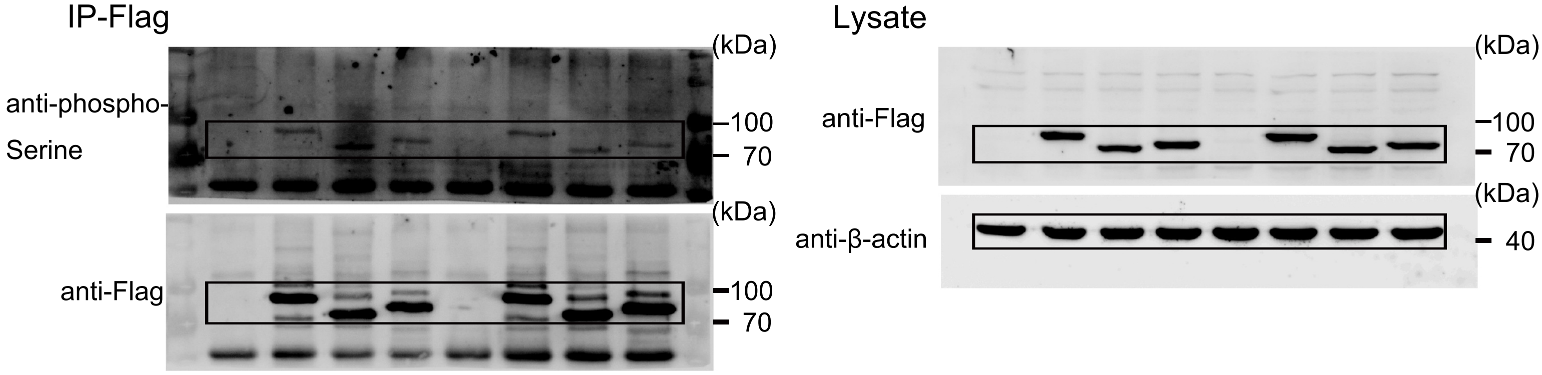
IP-Flag
(kDa)
anti-phospho-
Serine
100
70
(kDa)
anti-Flag
100
70
Lysate
(kDa)
anti-Flag
100
70
(kDa)
anti-β-actin
40

Supplementary Figure 12-16: Full-scan images of representative Western blots, reffered to Supplementary Figure 2-6

S2a

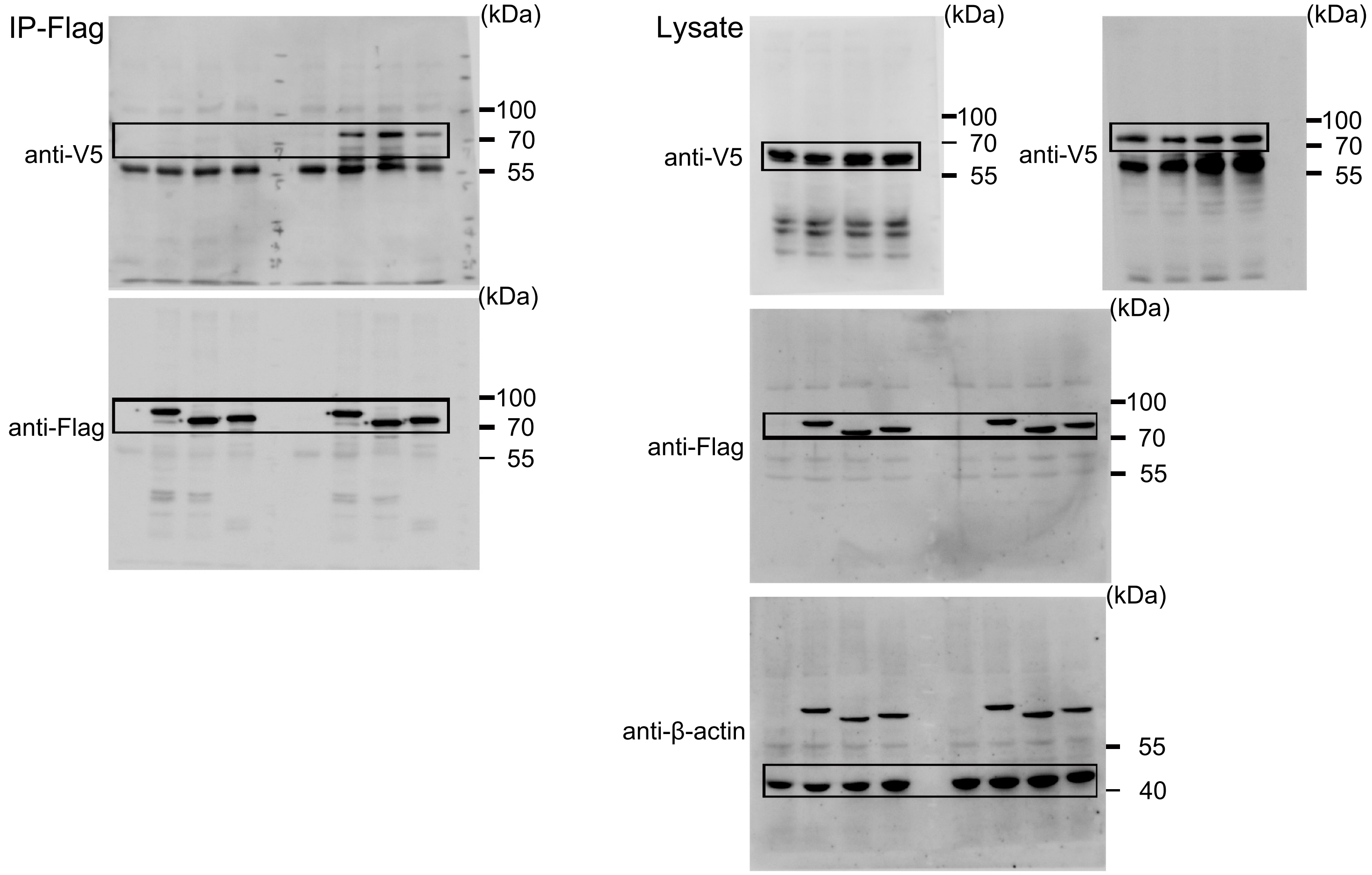
IP-Flag
(kDa)
anti-V5
100
70
55
(kDa)
anti-Flag
100
70
55
Lysate
(kDa)
anti-V5
100
70
55
(kDa)
anti-V5
100
70
55
(kDa)
anti-Flag
100
70
55
(kDa)
anti-β-actin
55
40

S3

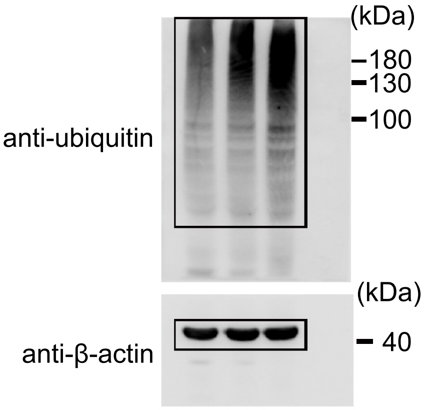

S4a

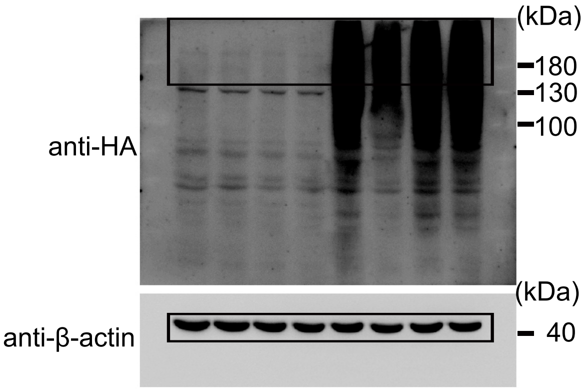

S4b

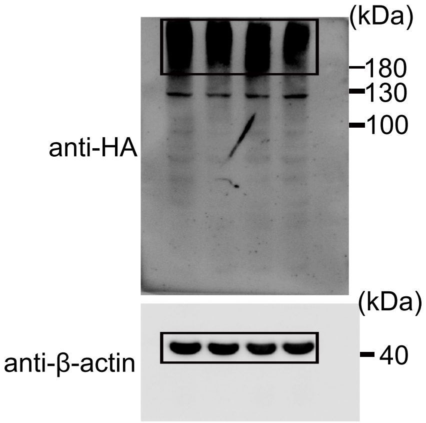

S4c

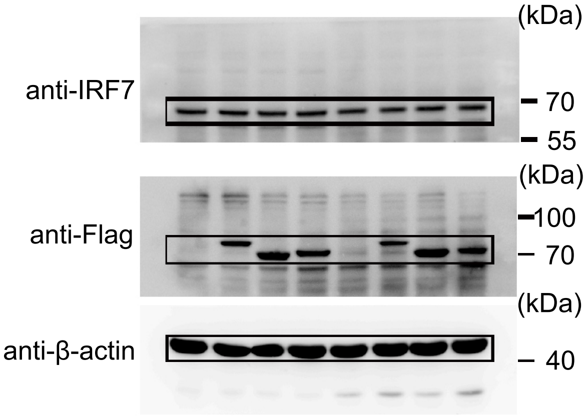

S4d

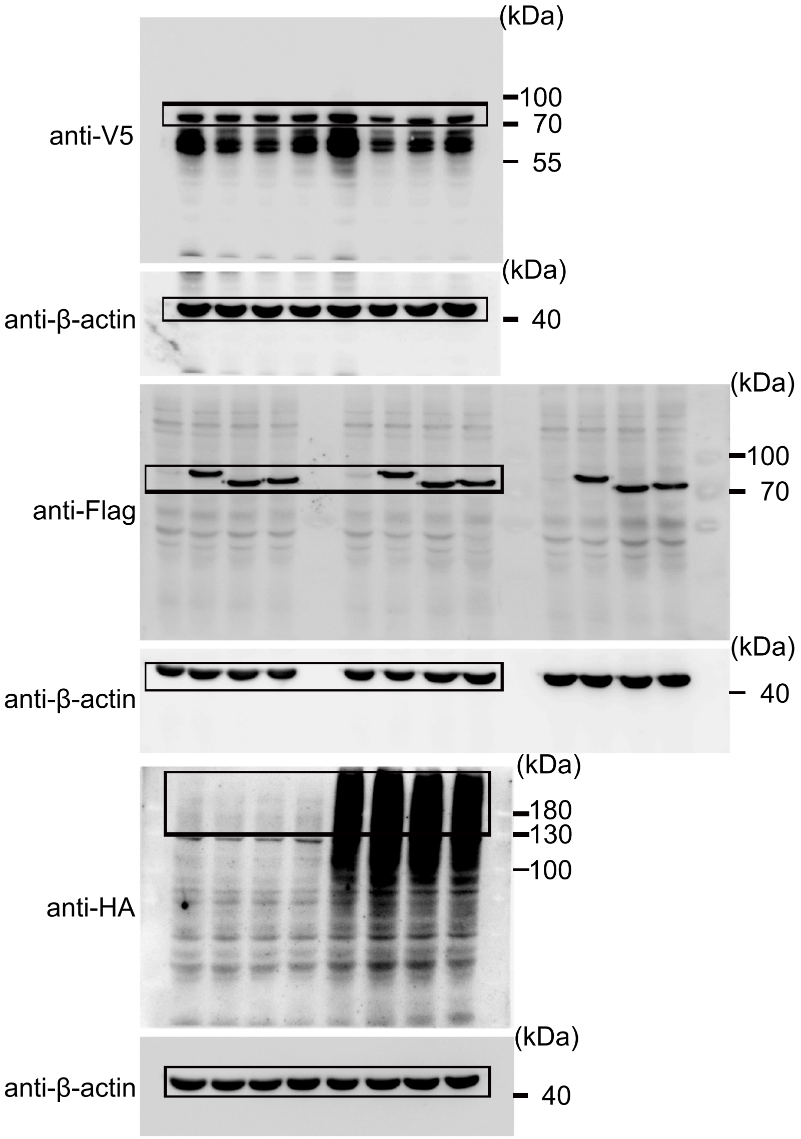

S4e

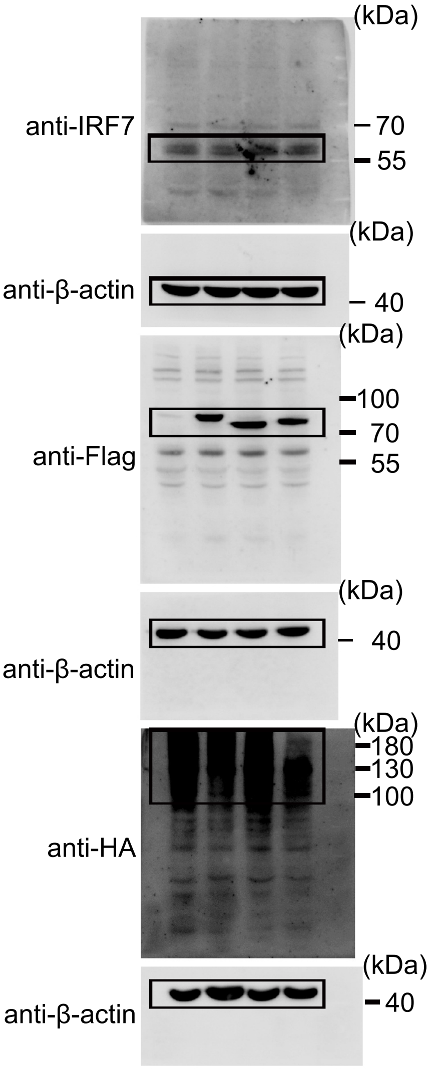

S5a

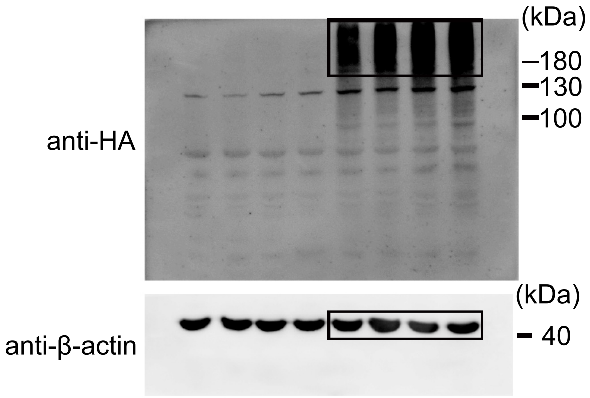

S5b

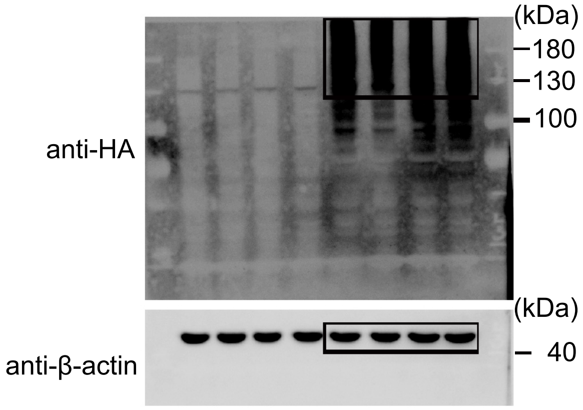

S5c

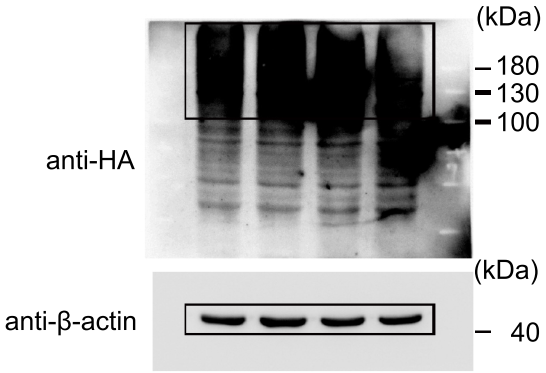

S5d

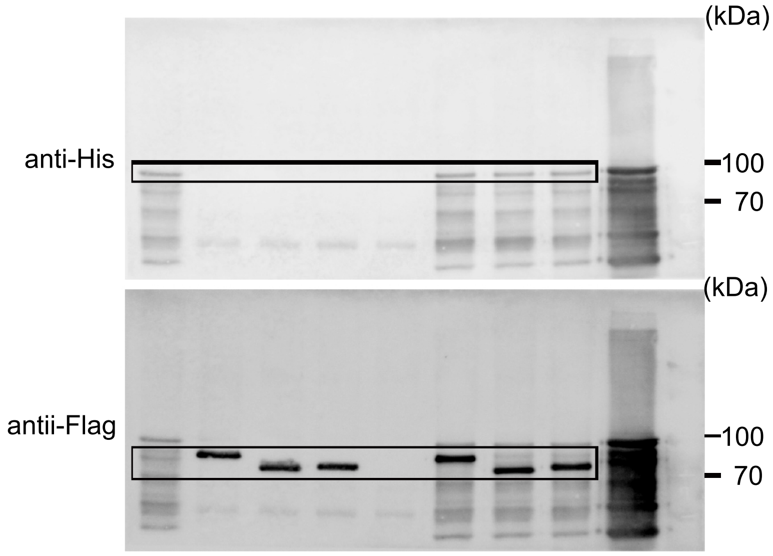

S6a

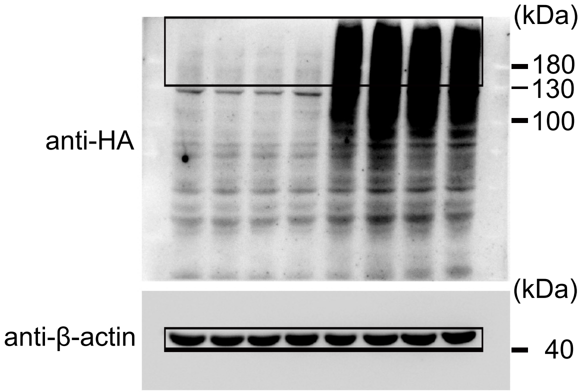
(kDa)
180
130
100
anti-HA
(kDa)
anti-β-actin
40

S6b

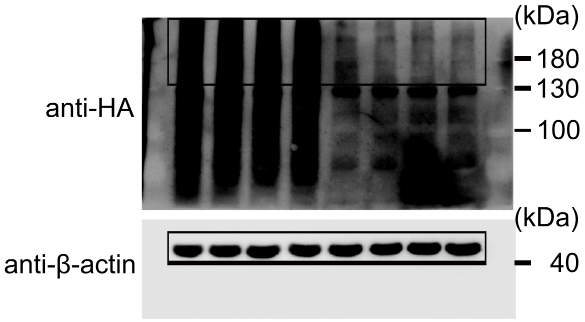
(kDa)
180
130
100
anti-HA
(kDa)
anti-β-actin
40

S6c

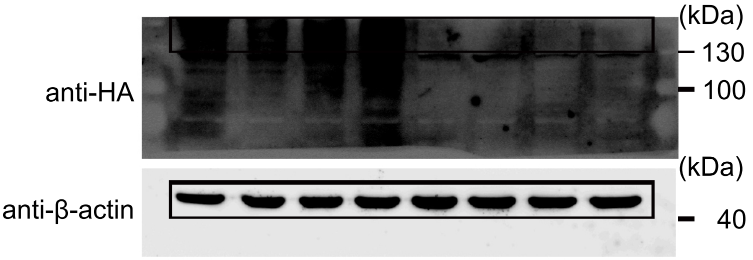
(kDa)
130
100
anti-HA
(kDa)
anti-β-actin
40
