# Supplementary material for: IKKε isoform switching governs the immune response against EV71 infection
Source: Commun Biol. 2021 Jun 2;4:663. doi: 10.1038/s42003-021-02187-x (PMC8172566; doi:10.1038/s42003-021-02187-x)
Supplement: Supplementary file 3 — Description of Supplementary Files [file 42003_2021_2187_MOESM3_ESM.pdf]

Description of Additional Supplementary Files

File name: Supplementary Data

Description: The Source data tab provides a legend for interpreting the source data presented in 'Supplementary data tabs 1-12' within the excel file.
